# Supplementary material for: New Preparative Approach to Purer Technetium-99 Samples—Tetramethylammonium Pertechnetate: Deep Understanding and Application of Crystal Structure, Solubility, and Its Conversion to Technetium Zero Valent Matrix
Source: Int J Mol Sci. 2023 Jan 19;24(3):2015. doi: 10.3390/ijms24032015 (PMC9916763; doi:10.3390/ijms24032015)
Supplement: Supplementary file 1 [file ijms-24-02015-s001.zip › ijms-2139441-supplementary.pdf]

Supporting Information

New preparative approach to purer technetium-99 samples. Tetramethylammonium pertechnetate: deep understanding and application of crystal structure, solubility, and its conversion to technetium zero valent matrix

Mikhail A. Volkov[1*], Anton P. Novikov[1,2], Mikhail S. Grigoriev[1], Vitaly V. Kuznetsov[1,3], Anastasiia V. Sitanskaya[1], Elena V. Belova[1], Andrew V. Afanasiev[1], Yuri M. Nevolin[1], Konstantin E. German[1]

[1] Frumkin Institute of Physical Chemistry and Electrochemistry, Russian Academy of Sciences, 31 Bldg 4, Leninsky prosp., Moscow, 119071, Russian Federation
[2] Peoples' Friendship University of Russia (RUDN University), 6 Miklukho-Maklaya Street, Moscow, 117198, Russian Federation
[3] Mendeleev University of Chemical Technology, 9 Miusskaya Square, Moscow, 125047, Russian Federation
* Correspondence: mikhailalexvol@gmail.com

Table S1. Bond Lengths for **1.**

Atom	Atom	Length/Å		Atom	Atom	Length/Å
Tc1	O1	1.665(3)		N1	$C1^2$	1.479(3)
Tc1	$O1^1$	1.665(3)		N1	C1	1.479(3)
Tc1	O2	1.689(4)		N1	$C2^2$	1.481(3)
Tc1	O3	1.693(4)		N1	C2	1.481(3)

^{1}+X,+Y,3/2-Z; ^{2}+X,1/2-Y,1-Z

Table S2. Bond Angles for **1.**

Atom	Atom	Atom	Angle/°		Atom	Atom	Atom	Angle/°
O1	Tc1	$O1^1$	114.1(4)		$C1^2$	N1	C1	110.3(3)
O1	Tc1	O2	107.1(2)		$C1^2$	N1	C2	109.3(2)
$O1^1$	Tc1	O2	107.1(2)		C1	N1	C2	108.7(2)
O1	Tc1	O3	109.46(15)		$C1^2$	N1	$C2^2$	108.7(2)
$O1^1$	Tc1	O3	109.46(15)		C1	N1	$C2^2$	109.3(2)
O2	Tc1	O3	109.5(2)		$C2^2$	N1	C2	110.4(3)

^{1}+X,+Y,3/2-Z; ^{2}+X,1/2-Y,1-Z

Table S3. Bond Lengths for **2.**

Atom	Atom	Length/Å		Atom	Atom	Length/Å
Re1	$O1^1$	1.665(7)		N1	C1	1.475(8)
Re1	O1	1.665(7)		N1	$C1^2$	1.475(8)

Re1	O2	1.686(9)		N1	C2[2]	1.480(8)
Re1	O3	1.714(8)		N1	C2	1.480(8)

[1]+X,+Y,3/2-Z; [2]+X,1/2-Y,1-Z

Table S4. Bond Angles for **2.**

Atom	Atom	Atom	Angle/°		Atom	Atom	Atom	Angle/°
O1[1]	Re1	O1	113.2(9)		C1	N1	C1[2]	109.8(8)
O1[1]	Re1	O2	107.5(4)		C1[2]	N1	C2[2]	109.1(5)
O1	Re1	O2	107.5(4)		C1	N1	C2[2]	109.1(5)
O1[1]	Re1	O3	108.9(3)		C1	N1	C2	109.1(5)
O1	Re1	O3	108.9(3)		C1[2]	N1	C2	109.0(5)
O2	Re1	O3	110.7(4)		C2	N1	C2[2]	110.8(8)

[1]+X,+Y,3/2-Z; [2]+X,1/2-Y,1-Z

Table S5. Bond Lengths for **3.**

Atom	Atom	Length/Å		Atom	Atom	Length/Å
Re1	O1	1.633(10)		C13	C14	1.371(11)
Re1	O2	1.628(11)		C21	C22	1.426(10)
Re1	O3	1.699(12)		C22	C23	1.415(11)
Re1	O4	1.644(9)		C23	C24	1.374(11)
N1	C11	1.564(16)		C31	C32	1.405(10)
N1	C21	1.47(2)		C32	C33	1.386(10)
N1	C31	1.61(3)		C33	C34	1.372(11)
N1	C41	1.473(13)		C41	C42	1.366(11)
C11	C12	1.353(11)		C42	C43	1.370(11)
C12	C13	1.361(9)		C43	C44	1.371(12)

Table S6. Bond Angles for **3.**

Atom	Atom	Atom	Angle/°		Atom	Atom	Atom	Angle/°
O1	Re1	O3	107.0(10)		C12	C11	N1	123.2(15)
O1	Re1	O4	112.6(6)		C11	C12	C13	134.9(15)
O2	Re1	O1	112.7(9)		C12	C13	C14	133.3(14)
O2	Re1	O3	107.3(6)		C22	C21	N1	117.7(10)
O2	Re1	O4	111.1(9)		C23	C22	C21	121.0(12)
O4	Re1	O3	105.6(9)		C24	C23	C22	126.5(16)
C11	N1	C31	104.1(17)		C32	C31	N1	117.8(11)
C21	N1	C11	111.6(10)		C33	C32	C31	126.6(12)
C21	N1	C31	110.3(8)		C34	C33	C32	129.6(14)
C41	N1	C11	111.3(7)		C42	C41	N1	131.0(15)
C41	N1	C21	111.5(19)		C41	C42	C43	131.5(17)

C41	N1	C31	107.8(12)		C42	C43	C44	131.2(18)

Table S7. Torsion Angles for **3.**

A	B	C	D	Angle/°		A	B	C	D	Angle/°
N1	C11	C12	C13	-162.3(17)		C21	N1	C41	C42	-169.0(15)
N1	C21	C22	C23	158(3)		C21	C22	C23	C24	166(4)
N1	C31	C32	C33	-164(2)		C31	N1	C11	C12	-168.6(12)
N1	C41	C42	C43	-172(2)		C31	N1	C21	C22	51.0(19)
C11	N1	C21	C22	166.1(14)		C31	N1	C41	C42	69.8(18)
C11	N1	C31	C32	-65.6(16)		C31	C32	C33	C34	53(4)
C11	N1	C41	C42	-44(3)		C41	N1	C11	C12	-53(2)
C11	C12	C13	C14	-71(3)		C41	N1	C21	C22	-68.8(17)
C21	N1	C11	C12	72.5(16)		C41	N1	C31	C32	176.2(14)
C21	N1	C31	C32	54.2(18)		C41	C42	C43	C44	-176(3)

Table S8. Bond Lengths for **4.**

**Table 4 Bond Lengths for bu4n.**						
**Atom**	**Atom**	**Length/Å**		**Atom**	**Atom**	**Length/Å**
Tc1	O1	1.601(8)		C13	C14	1.284(8)
Tc1	O2	1.617(8)		C21	C22	1.283(8)
Tc1	O3	1.621(8)		C22	C23	1.285(8)
Tc1	O4	1.607(8)		C23	C24	1.283(8)
N1	C11	1.497(9)		C31	C32	1.287(7)
N1	C21	1.501(9)		C32	C33	1.290(7)
N1	C31	1.500(9)		C33	C34	1.295(7)
N1	C41	1.490(9)		C41	C42	1.285(7)
C11	C12	1.266(7)		C42	C43	1.284(7)
C12	C13	1.282(7)		C43	C44	1.284(7)

Table S9. Bond Angles for **4.**

**Table 5 Bond Angles for bu4n.**								
**Atom**	**Atom**	**Atom**	**Angle/°**		**Atom**	**Atom**	**Atom**	**Angle/°**
O1	Tc1	O2	110.4(6)		C12	C11	N1	149.9(10)
O1	Tc1	O3	110.0(6)		C11	C12	C13	151.5(14)
O1	Tc1	O4	111.6(5)		C12	C13	C14	142.8(18)
O2	Tc1	O3	107.4(5)		C22	C21	N1	107.6(13)
O4	Tc1	O2	109.0(6)		C23	C22	C21	143(2)
O4	Tc1	O3	108.3(6)		C24	C23	C22	143(2)
C11	N1	C21	108.5(6)		C32	C31	N1	128.8(12)
C11	N1	C31	107.5(6)		C31	C32	C33	141.7(14)
C31	N1	C21	108.7(6)		C32	C33	C34	138.6(14)

C41	N1	C11	111.0(5)		C42	C41	N1	133.0(12)
C41	N1	C21	110.5(6)		C43	C42	C41	146.8(16)
C41	N1	C31	110.5(6)		C42	C43	C44	145.3(17)

Table S10. Torsion Angles for **4.**

**Table 6 Torsion Angles for bu4n.**										
**A**	**B**	**C**	**D**	**Angle/°**		**A**	**B**	**C**	**D**	**Angle/°**
N1	C11	C12	C13	-105(4)		C21	N1	C41	C42	-161.7(14)
N1	C21	C22	C23	155(4)		C21	C22	C23	C24	76(11)
N1	C31	C32	C33	-166(3)		C31	N1	C11	C12	-153(2)
N1	C41	C42	C43	-138(4)		C31	N1	C21	C22	60.2(19)
C11	N1	C21	C22	176.8(18)		C31	N1	C41	C42	77.9(16)
C11	N1	C31	C32	-71(2)		C31	C32	C33	C34	49(6)
C11	N1	C41	C42	-41.3(16)		C41	N1	C11	C12	-32(2)
C11	C12	C13	C14	-162(5)		C41	N1	C21	C22	-61.3(19)
C21	N1	C11	C12	89(2)		C41	N1	C31	C32	168(2)
C21	N1	C31	C32	46(2)		C41	C42	C43	C44	-135(5)
